# How the Soil Microbial Communities and Activities Respond to Long-Term Heavy Metal Contamination in Electroplating Contaminated Site

**DOI:** 10.3390/microorganisms9020362

**Published:** 2021-02-12

**Authors:** Wen-Jing Gong, Zi-Fan Niu, Xing-Run Wang, He-Ping Zhao

**Affiliations:** 1 MOE Key Lab of Environmental Remediation and Ecosystem Health, College of Environmental and Resource Sciences, Zhejiang University, Hangzhou 310058, China; gongwenjing@zju.edu.cn (W.-J.G.); zfniu@zju.edu.cn (Z.-F.N.); 2 Chinese Research Academy of Environmental Sciences, Beijing 100012, China; wangxr@craes.org.cn

**Keywords:** heavy metals, soil enzyme, microbial biomass carbon, microbial community structure

## Abstract

The effects of long-term heavy metal contamination on the soil biological processes and soil microbial communities were investigated in a typical electroplating site in Zhangjiakou, China. It was found that the soil of the electroplating plant at Zhangjiakou were heavily polluted by Cr, Cr (VI), Ni, Cu, and Zn, with concentrations ranged from 112.8 to 9727.2, 0 to 1083.3, 15.6 to 58.4, 10.8 to 510.0 and 69.6 to 631.6 mg/kg, respectively. Soil urease and phosphatase activities were significantly inhibited by the heavy metal contamination, while the microbial biomass carbon content and the bacterial community richness were much lower compared to noncontaminated samples, suggesting that the long-term heavy metal contamination had a severe negative effect on soil microorganisms. Differently, soil dehydrogenase was promoted in the presence of Chromate compared to noncontaminated samples. This might be due to the enrichment of *Sphingomonadaceae*, which have been proven to be able to secrete dehydrogenase. The high-throughput sequencing of the 16S rRNA gene documented that *Proteobacteria*, *Actinobacteria*, and *Chloroflexi* were the dominant bacterial phyla in the contaminated soil. The Spearman correlation analysis showed the *Methylobacillus*, *Muribaculaceae*, and *Sphingomonadaceae* were able to tolerate high concentrations of Cr, Cr (VI), Cu, and Zn, indicating their potential in soil remediation.

## 1. Introduction

Heavy metals (HMs) are severe pollutants in soil environments from either natural processes or anthropogenic activities [1]. Electroplating industrial plants are the most common sources of toxic metals by direct or indirect discharge, though they play an important role in the Chinese economy [2,3]. The heavy metal waste usually piled up in large quantities, causing a potential threat to the safety of groundwater, soil, and the atmospheric environment [4]. Among them, copper (Cu), zinc (Zn), nickel (Ni), and chromium (Cr) were most common in soil. Thus, the identification of HMs pollution is of significant ecological and anthropic importance.

Soil microbial enzyme activities (EAs) and microbial biomass carbon (MBC), are sensitive to soil ecosystem disturbance [5]. EAs and MBC can reflect the direction and degree of soil biochemical reactions, and serve as potential biological indicators to diagnose soil health [6]. Among enzymes, urease (UA), phosphatase (PHA), and dehydrogenase (DHA) are often used as HMs enzyme markers [5].

Soil microbial community structure and diversity are also highly sensitive to soil environmental changes and are often used as indicators of metal contaminations as well [6]. Many studies have shown the evolution of the microbial community caused by HMs under long-term contamination [7,8,9]. Song [10] found that the microbial communities changed with HMs (Cd, Cu, Zn) concentrations and soil physicochemical properties (pH, TN, TC): while Li [11] found that Cr, Pb, and Zn all negatively affected the abundance of *Nitrospirae*, *Bacteroidetes*, and *Verrucomicrobia*. However, there have been few studies on how the HMs’ combined pollution affects microbial community structure, EAs, and MBC at electroplating sites.

In this study, an abandoned electroplating site from Zhangjiakou (Hebei Province, China) was selected for further analysis. The soil environmental parameters, including the concentrations of Cr, Cr (VI), Ni, Cu, and Zn, were measured. Specifically, the aims of this study were: (i) to explore the relationships among soil HMs contamination, physicochemical properties, soil EAs, MBC, and the structures of the microbial community at in situ electroplating sites; (ii) to predict the possible functions of dominant bacteria in the process of ecological restoration.

## 2. Materials and Methods 

### 2.1. Study Area

The Electroplating Plant at Zhangjiakou (EPZ, 40°50′12.008″ N, 114°52′26.451″ E) which was used for production since the 1960s, ceased production in 2015. The EPZ lies in the temperate continental monsoon climate zone with an annual mean temperature of 9.6 °C, and multiyear average precipitation of about 350 mm [12,13]. The EPZ includes six electroplating processes located in different areas. The six processes have different HMs concentrations theoretically (Figure S1): location 1 was an electroplating laboratory (S0); location 2 was a chrome bath (S1), location 3 was a decorative chrome tank (S2); location 4 was a postplating treatment room (S3); location 5 was a galvanizing bath (S4), while location 6 was a sewage treatment tank (S5). To reveal the in situ HMs concentration as accurately as possible, we took five samples from different depths in each location. Besides, for comparison with the local soil background value, we collected noncontaminated soil 5 km away from the plant.

### 2.2. Soil Collection

Thirty soil samples were collected in November 2019 and carried back to the laboratory within 12 h at 4 °C. All soil samples were divided into two groups, one for molecular genome experiment (stored at −20 °C), another was for physicochemical properties and HMs measurement (air-dried and processed with a 2 mm sieve to remove the stone and root fragments, stored at 4 °C) [14].

### 2.3. Soil Physicochemical and HMs

The pH was tested in deionized water at a soil/solution ratio of 1:2.5 using HI 3221 pH meter from a 2 mm soil fraction (Seven Easy, Mettler-Toledo, Switzerland). Total contents of Cr/Ni/Cu/Zn in the soil were analyzed with TAS-990 flame atom adsorption spectrophotometer (Persee Inc., Beijing, China) by digesting 100 mg of soil in a mixture of HCl-HNO_3_-HF-HClO_4_, which was constituted with 3 mL of 1.19 g/mL HCl, 5 mL of 1.42 g/mL HNO_3_, 5 mL of 1.49 g/mL HF, 3 mL of 1.68 g/mL HClO_4_ and finally constant volume to 50 mL using deionized water. The digestion program was processed by two stages of heating: 30 min at 120 °C and 1 h at 200 °C (Persee Inc., Beijing, China). Besides, the total Cr (VI) concentration of soil samples was measured by alkaline digestion standard method, using a mixture of 50 mL NaCO_3_/NaOH mixed solution, 400 mg MgCl_2_, and 50 mL K_2_HPO_4_-KH_2_PO_4_, and heat at 90–95 °C for 60 min [15].

### 2.4. Microbial Biomass Carbon and Soil EAs

Microbial biomass carbon (MBC) was determined by the fumigation-extraction (FE) method [16]. PHA was measured spectrophotometrically by the disodium phenyl phosphate method of Li [17] by measuring the phenolic micrograms per gram of soil. UA activity was determined by the indophenol colorimetric method by measuring NH_4_^+^-N generated per gram of soil after 24 h [18]. DHA was tested by reduction of 2,3,5-triphenyltetrazolium chloride (TTC) method [5].

### 2.5. DNA Extraction and Sequencing

In this study, we analyzed the bacterial community structure in all soil samples using the Illumina MiSeq PE300 high-throughput sequencing approach. Total genomic DNA was extracted from 0.25 g of well-mixed soil for each sample using the Power Soil^®^ DNA Isolation Kit (Qiagen, Redwood City, US Functional Area, USA) following the manufacturer’s protocol described by Zhao [19,20,21]. DNA concentration and purity were determined with microspectrophotometry (NanoDrop^®^). Extracted DNA was sent to Mingke Biotechnology (Hangzhou) Co., Ltd, amplified with 341F (5′-CCTAYGGGRBGCASCAG-3′) and 806R (5′-GGACTACNNGGGTATCTAAT-3′) [22,23]. The purified amplicons were then sequenced on an Illumina Miseq sequencing platform. Sequences with >97% similarity were assigned to the same operational taxonomic units (OTUs). Representative sequences of each OTU were screened for further annotation [10]. Besides, the *α*-diversity of the bacterial community was determined by the Chao, Shannon, and Simpson indices, and the number of observed species was generated by QIIME2 [24,25].

### 2.6. Data Analysis

The correlation analysis of soil physicochemical, HMs, and microbial activities were completed with the corrplot R package (250 Northern Ave, Boston, MA 02210). Spearman correlation coefficient was calculated with SPSS. Other plots were completed using software Origin (1 Roundhouse Plaza, Northampton, MA, USA).

## 3. Results and Discussion

### 3.1. Soil HMs Contamination Level and Physicochemical Properties

The HMs concentrations of tested soil samples are shown in Table 1. The concentration of total Cr ranged from 112.8 to 9727.2 mg/kg with an average of 1617.3 mg/kg, while Cr (VI) ranged from 0 to 1083.3 mg/kg (average value 111.1 mg/kg), Ni varied from 15.6 to 58.4 mg/kg with mean value of 30.5 mg/kg, Cu and Zn ranged from 10.8 to 510.0 mg/kg and 69.6 to 631.6 mg/kg with an average of 105.5 mg/kg and 186.8 mg/kg, respectively. Clearly, the concentrations of each HMs were closely related to the electroplating process. For example, the concentrations of Cr and Cr (VI) were far higher in S1 (chrome bath) and S2 (decorative chrome tant) (Figure S1) than at the other sites. According to a risk control standard for soil contamination of developing land (GB36600-2018) [26], the concentration of Cr (VI) severely exceeded the standard (3 mg/kg) at most sites, while Cu and Ni did not exceed the standard (Cu: 2000 mg/kg, Ni: 150 mg/kg). The screening value of Zn differs from province to province, and it is 700 mg/kg in Hunan province [27]. Thus, Cr pollution became the main contamination in EPZ.

**Figure 1:**
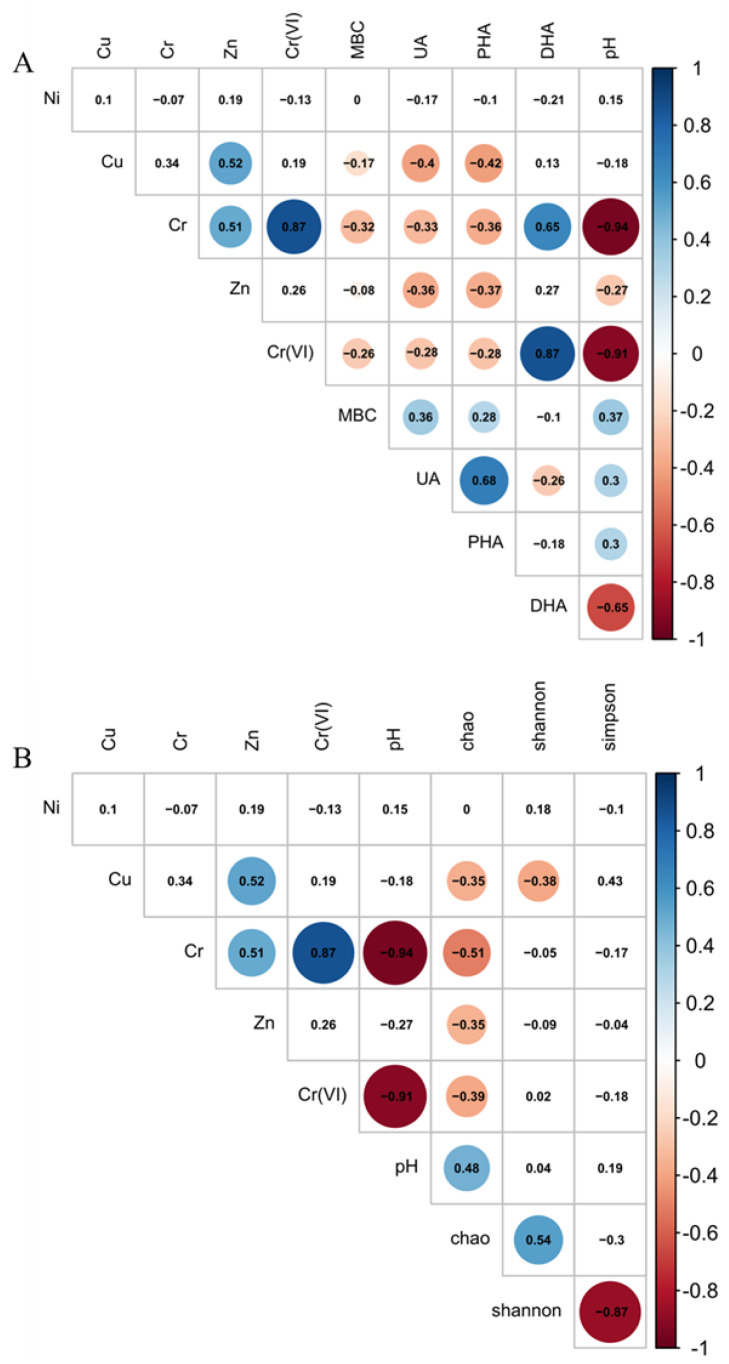
The correlation analysis between the environmental factors and soil microbial activities (**A**) and α-diversity indexes (**B**) of soil. (Digits represent Pearson correlation coefficients; filled color indicates significant correlation, blue represents positive correlation, red represents negative correlation, shade of color represents strength of correlation).

The soil pH is shown in Table S1. Almost all soil samples were alkaline, except the S2 sites (lower pH ranged from 4.24 to 6.23), which contained more Cr and Cr (VI) than the other sites. Theoretically, as shown in Equation (1), soluble Cr (VI) will be converted to dissoluble Cr (III) by H^+^ under acid conditions, and with the decrease in pH, more precipitated Cr (III) is generated [28,29]. However, the pH was negatively correlated with the concentrations of Cr and Cr (VI) in this study (Cr, r = −0.94, *p* < 0.01; Cr (VI), r = −0.91, *p* < 0.01) through Pearson Correlation Analysis (Figure 1). Taking human factors into consideration, as shown in Equation (2), sulfuric acid and potassium dichromate were used in the decorative chrome plating process (heating to generate chromium trioxide, which was the raw material), the leakage of which may cause a lower pH and higher Cr (VI). The negative correlation between pH and Zn (r = −0.27, *p* < 0.05) concentration is partly due to the adsorption of Zn^2+^ on oxides and aluminosilicates, which reduced its mobility around pH 7. The formation or dissolution of a organic Zn complex at high pH will weaken the pH effects on Zn concentration, but it will not reverse the correlation [30].

Cr_2_O_7_^2−^ + 8H^+^ + 6e^−^ = 2Cr(OH)_3_ + H_2_O (1)

2H_2_SO_4_ + Na_2_Cr_2_O_7_ = 2 CrO_3_ + 2 NaHSO_4_+ H_2_O (2)

Meanwhile, pH was positively correlated with MBC, UA, and PHA (MBC, *r* = 0.37, *p* < 0.05; UA, *r* = 0.3, *p* < 0.05; PHA, *r* = 0.3, *p* < 0.05), and negatively correlated with DHA (*r* = −0.65, *p* < 0.01). The same result was reported by Wic Baena and Jorge-Mardomingo [31,32]: pH had a positive relationship with UA (*r* = 0.33, *p* < 0.001) and PHA (*r* = 0.43, *p* < 0.001) and a negative relationship with DHA (*r* = −0.45, *p* < 0.001). As shown in Table 2, the pH was positively correlated with the abundance of *Arthrobacter* (*r* = 0.40, *p* < 0.05), which have been proved highly active in an alkaline environment [33]. Members of the genus *Arthrobacter* were found able to secret PHA and UA [34,35]: the positive relationship between pH, UA, and PHA might be due to the enrichment of *Arthrobacter* in alkaline environments, thus more PHA and UA were produced. 

### 3.2. Relationship Between HMs, EAs, and MBC

HMs had a strong influence on the soil EAs and MBC [36]. The EAs concentrations in the soils at different sites were shown in Table S1. As shown in Figure 1A, the EAs and MBC were negatively correlated with nearly all HMs in the electroplating plant.

DHA was very sensitive to HMs contamination and was usually used as an indicator of chromium contamination in soil [37,38,39,40]. In this study, DHA was positively correlated with the Cr and Cr (VI) concentration (Cr, *r* = 0.65, *p* < 0.01; Cr (VI), *r* = 0.87, *p* < 0.01). However, previous studies showed that Cr(VI) can inhibit DHA activity by over 70% after 35 d, and DHA activities were the lowest with 0.25 g/kg Cr (VI) [39]. Furthermore, the increase of chromium (K_2_Cr_2_O_7_) from 40 mg/kg to 120 mg/kg caused a decrease of DHA from 2.90 U/g to 0.25 U/g [40]. Thus, we assumed that other factors like microbial processes might affect the activity of DHA.

As shown in Table 2, the abundance of *Sphingomonadaceae_uncultured* increased with Cr and Cr (VI) concentration (Cr, *r* = 0.463, *p* < 0.05; Cr (VI), *r* = 0.738, *p* < 0.01). Since the *Sphingomonadaceae* family were found to secret DHA [41,42], the positive relationship between DHA and Cr/Cr (VI) might be that Cr and Cr (VI) stimulated the growth of *Sphingomonadaceae* family which could produce DHA. 

PHA usually acts as a catalyst in the hydrolysis of ester and anhydride of phosphoric acid [29]. In present study, PHA was observed negatively correlated to all the metals except Ni (Cr, *r* = −0.36, *p* < 0.05; Cr (VI), *r* = −0.28, *p* < 0.05; Cu, *r* = −0.42, *p* < 0.05; Zn, *r* = −0.37, *p* < 0.05). The UA, which was considered to be closely associated with the transformation, biological turnover, and bioavailability of N [43], had a negative correlation with HMs (Cr, *r* = −0.33, *p* < 0.05; Cr (VI), *r* = −0.28, *p* < 0.05; Zn, *r* = −0.36, *p* < 0.05; Cu, *r* = −0.4, *p* < 0.05) too. Similar results found that Cr, Cr(VI), Cu, and Zn had negative correlations with PHA and UA in previous studies [14,44,45,46]. As shown in Table 2, the Cr and Cr (VI) had a negative correlation with *Methylophilaceae_Unclassified* and *Vicinamibacterales_norank*. Since they were found to secret PHA and UA [34,47,48,49], the negative relationship between PHA, UA, and Cr, Cr (VI) might be caused by the *Methylophilaceae* and *Vicinamibacterales* family members inhibited by Cr and Cr (VI).

The microbial biomass of soil is comprised by the total mass of fungi, bacteria, protozoa, and algae [50]. It is usually correlated with the supply of carbon (C) substrate [51]. MBC represents the microbial community size and usually decreased by HMs. Thus, MBC has frequently been used to investigate the long-term impact of HMs on microorganisms within the soil environment [46,52,53,54]. In our study, MBC was negatively correlated with Cu, Cr, and Cr (VI) (Cu, *r* = −0.17, *p* < 0.05; Cr, *r* = −0.32, *p* < 0.05, Cr (VI), *r* = −0.26, *p* < 0.05), and decreased compared to the noncontaminated soils (Table S1). The reason might be that under the HMs pressure, microorganisms spend more energy on detoxification rather than growth and biomass accumulation [55].

### 3.3. Relationship between Bacterial Diversity and HMs 

The Illumina MiSeq platform was used to analyze the bacterial diversity of the soil samples across the six contaminated sites. A total of 1,348,872 16S rDNA trimmed sequences with an average length of 416 bp were obtained for classification. The sequences were placed into 60–1778 operational taxonomic units (OTUs) at a level of sequence similarity of ≥97%. To compare species richness, rarefaction curves were generated by randomly sampling reads and plotting the number of novel 97% OTUs against the number of sample size (Figure S2). Clearly, the increase in sample size contributed to an increase in OTUs. As the sample size increased, the curve tended to be flat, indicating a sufficient sequencing data volume.

The bacterial α-diversity index is shown in Table S2. It had been reported that long-term contamination with high load HMs (Cr, Cu and Zn) could shift the abundance and the diversity of microbial communities [56]. The Chao indices represent the microbial richness and was negatively related to Cu, Cr, Zn, and Cr (VI) (Cu, *r* = −0.35, *p* < 0.05; Cr, r = −0.51, *p* < 0.01; Zn, r = −0.35, *p* < 0.05; Cr (VI), r = −0.39, *p* < 0.05) (Figure 1B) in this study. The same result showing a negative relationship between Cr and Chao was found in Li’s research [11], while there was no evidence supporting relationships among Ni, Cu, Zn, and Chao. Furthermore, the Shannon and Simpson indices represented the microbial diversity and only Shannon was negatively correlated with Cu (*r* = −0.38, *p* < 0.05). As illustrated by Kong, the addition of Cu up to 100 μm strongly reduced functional diversity and evenness of microbial community, suggesting that Cu was the key factor in reducing microbial diversity [57]. Besides, the bacterial community’s richness and diversity decreased significantly compared to the noncontaminated soils. Previous studies showed that the diversity of sensitive microbial species abruptly decreased under long-term HMs contamination, while the abundance of resistant microorganisms increased by adapting to new habitats [56,58,59]. This further supported the assumption that the growth of microorganisms was inhibited and the sensitive species might be replaced by resistant species under the pressure of HMs, causing a lower microbial richness and diversity.

### 3.4. Relationship between Bacterial Community and HMs

**Figure 2:**
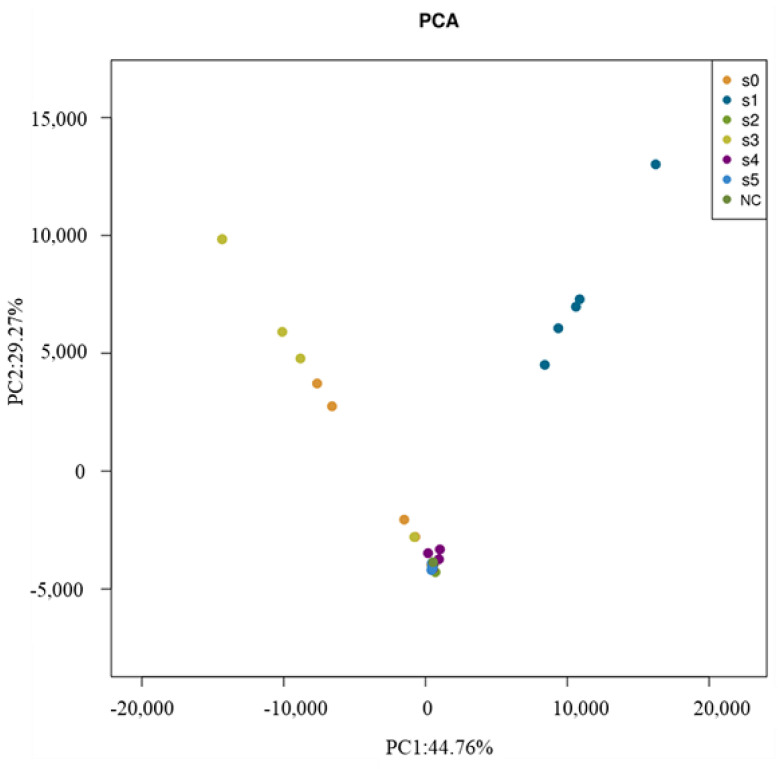
Principal component analysis for the bacterial 16S rDNA sequences. (NC: noncontaminated soil).

By using principal component analysis (PCA), we separated the data of bacterial genus into two factors that explained 74.03% of the variance (Figure 2). Clearly, the bacterial communities of the S1 site, which contained more *Methylobacillus*, were totally different from other sites. The other sites (S0, S2, S3, S4 and S5) were grouped tightly, indicating that most of the soil samples shared high similarity in terms of their bacterial structure.

**Figure 3:**
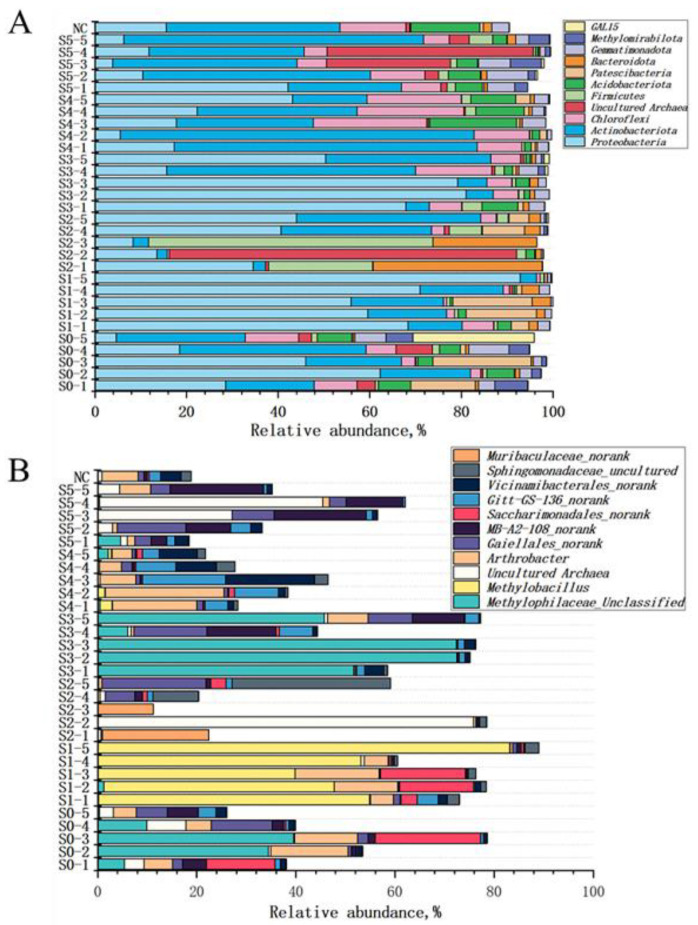
Community composition of bacteria phylum (**A**) and genus (**B**) in the soil. (NC: noncontaminated soil).

For further analysis, the bacterial community composition of contaminated soils was investigated, and the result is shown in Figure 3A at the phylum level and Figure 3B at the genus level. The phylum shown in the Figure 3A is based on the taxa with a total abundance >30% of all samples. Besides, the genus shown in the Figure 3B is based on the taxa with a total abundance >30% of all samples, and abundance >10% in at least one sample. The *Actinobacteria* and *Proteobacteria* were the dominant phyla in all samples except S2-2 and S2-3, while *Chloroflexi*, *Firmicutes*, *Acidobacteria*, *Patescibacteria*, and *Gemmatimonadota* accounted for a small amount proportion. At the genus level, the *Methylophilaceae_Unclassified, Methylobacillus, Arthrobactery* and *Muribaculaceae_norank*, belonged to *Proteobacteria* and *Actinobacteria* dominated the microbial communities in soil samples S0, S1, S2, S3 and S4. Besides, the *Actinobacteria* was dominant in the noncontaminated soils, while the *Proteobacteria* was dominant in the contaminated soils. Similarly, Sheik found that the contaminated soils all had a similar phylum-level abundance, and *Proteobacteria* was the dominant phylum in Cr contaminated soils in contrast to the control soils where *Actinobacteria* was the dominant phylum [60]. This suggested that the genus that belonged to *Proteobacteria* had more resistance towards HMs than that belonging to *Actinobacteria*.

The effects of HMs on bacteria community structure have been widely studied previously [61,62,63]. In this study, we investigated the relationships between HMs and some bacterial genus in EPZ soil samples, as shown in Table 2. We found that *Sphingomonadaceae_uncultured* was positively correlated with Cr (*r* = 0.406, *p* < 0.05), Zn (*r* = 0.574, *p* < 0.05) and Cr (VI) (*r* = 0.456, *p* < 0.01). 

Downstream genes *oscA*, which were found to resistant to Cr (VI), were proved to exist in *Methylobacillus*, suggesting that *Methylobacillus* may be related to Cr (VI) resistance [64]. Zhou reported that the abundance of *Sphingomonadaceae* increased with CrO_4_^2−^ exposure and became the major bacteria in the cake layer of a membrane bioreactor [65]. Liu showed that *Sphingomonadaceae* was significantly enriched at an 80 cm depth layer in chromium-contaminated soil [66]. Besides the *Sphingomonadaceae* was found dominated in many soils polluted with Zn, Cr and related to the Zn hyperaccumulation in the root [63,67,68], suggesting that *Sphingomonadaceae* might have a resistance to Cr, Cr (VI) and Zn, and might help Cr (VI) contaminated soil to recover ecological function. There were few research pieces focusing on the *Muribaculaceae*, and our result showed a positive correlation between *Muribaculaceae* and HMs, suggesting that it might have a strong resistance towards Cr, Cr (VI), and Cu, which had never been found before.

## 4. Conclusions

This study showed that DHA was significant positively correlated with Cr and Cr (VI) in contaminated soil, which had never been found in previous studies. High-throughput sequencing results verified that *Sphingomonadaceae*, which had been proved to be able to secrete DHA, was enriched in Cr contaminated soils. Meanwhile, the microorganism might divert energy from growth to cell maintenance functions under HMs pressure, causing a decrease in the richness of the bacterial community and MBC. Besides, *Methylobacillus* and *Muribaculaceae* might have strong resistance to Cr (VI), Zn, and Cu meaning these microbes might have advantages and potential applications in the bioremediation of heavy metal compound contaminated soils.

## Figures and Tables

**Table 1:** The heavy mental contents from different sampling sites.

	Total Ni (mg/kg)	Total Cu (mg/kg)	Total Cr (mg/kg)	Total Zn (mg/kg)	Total Cr (VI) (mg/kg)
S0-1	24.8 ± 0.72	38.0 ± 1.04	411.2 ± 0.72	83.6 ± 1.20	1.4 ± 0.28
S0-2	22.0 ± 0.40	20.0 ± 0.28	172.4 ± 0.44	83.6 ± 0.52	3.2 ± 0.34
S0-3	28.0 ± 0.16	29.2 ± 0.72	232.4 ± 0.80	105.6 ± 0.56	1.3 ± 0.37
S0-4	28.0 ± 0.52	20.0 ± 0.04	292.0 ± 0.68	91.2 ± 0.16	2.2 ± 0.22
S0-5	34.0 ± 1.52	35.2 ± 0.28	292.0 ± 0.20	124.8 ± 0.36	5.0 ± 0.64
S1-1	30.8 ± 0.56	16.8 ± 0.36	292.0 ± 0.20	96.4 ± 0.60	6.5 ± 0.68
S1-2	15.6 ± 0.20	16.8 ± 0.16	292.0 ± 0.36	143.6 ± 0.40	4.2 ± 0.20
S1-3	24.8 ± 0.76	10.8 ± 0.04	351.6 ± 0.44	69.6 ± 0.60	13.0 ± 0.22
S1-4	30.8 ± 0.72	486.0 ± 0.60	5786.0 ± 7.76	590.4 ± 0.52	104.5 ± 2.60
S1-5	24.8 ± 0.84	16.8 ± 0.16	351.6 ± 0.32	165.2 ± 0.16	82.0 ± 1.60
S2-1	18.8 ± 1.12	126.0 ± 0.08	7338.8 ± 4.76	258.0 ± 0.72	302.6 ± 1.04
S2-2	37.2 ± 0.40	132.0 ± 0.12	9727.2 ± 4.80	245.2 ± 2.40	861.9 ± 2.80
S2-3	28.0 ± 0.52	147.2 ± 0.28	9249.6 ± 6.44	251.2 ± 1.04	861.9 ± 3.40
S2-4	22.0 ± 0.40	219.6 ± 0.36	6681.6 ± 3.80	304.4 ± 0.60	1083.3 ± 4.40
S2-5	28.0 ± 0.52	23.2 ± 0.36	471.2 ± 0.24	133.2 ± 1.04	47.5 ± 1.04
S3-1	30.8 ± 0.32	258.8 ± 0.52	530.8 ± 0.20	156.0 ± 1.20	3.5 ± 0.24
S3-2	40.0 ± 0.28	376.8 ± 0.64	530.8 ± 0.24	191.6 ± 0.24	5.2 ± 0.04
S3-3	30.8 ± 0.24	510.0 ± 1.12	351.6 ± 0.24	121.6 ± 1.16	2.3 ± 0.04
S3-4	28.0 ± 0.20	86.4 ± 0.16	351.6 ± 0.52	98.8 ± 0.40	3.3 ± 0.68
S3-5	22.0 ± 0.76	20.0 ± 0.08	172.4 ± 0.44	123.2 ± 1.64	0.5 ± 0.44
S4-1	46.4 ± 0.56	189.2 ± 0.40	2859.6 ± 2.48	631.6 ± 2.08	0.1 ± 0.20
S4-2	28.0 ± 0.52	98.8 ± 0.12	590.4 ± 0.16	310.0 ± 2.60	23.3 ± 0.72
S4-3	40.0 ± 0.76	92.4 ± 0.24	590.4 ± 0.32	327.6 ± 0.88	1.8 ± 0.22
S4-4	22.0 ± 0.72	122.8 ± 0.76	351.6 ± 0.72	192.4 ± 0.88	7.4 ± 0.80
S4-5	30.8 ± 0.72	77.6 ± 3.48	411.2 ± 0.32	192.4 ± 0.40	8.4 ± 0.96
S5-1	24.8 ± 0.12	16.8 ± 0.20	112.8 ± 1.04	128.4 ± 2.08	3.5 ± 0.34
S5-2	30.8 ± 0.56	16.8 ± 0.16	292.0 ± 0.36	106.4 ± 1.80	1.0 ± 0.28
S5-3	37.2 ± 0.32	16.8 ± 0.08	351.6 ± 0.28	104.0 ± 0.20	2.1 ± 0.28
S5-4	58.4 ± 0.36	20.0 ± 0.04	232.4 ± 0.52	98.8 ± 0.40	0
S5-5	34.0 ± 0.52	10.8 ± 0.08	172.4 ± 0.80	148.4 ± 3.92	0
NC	43.2 ± 0.76	20.0 ± 0.03	292.0 ± 0.52	114.8 ± 0.19	0

Note: The results are presented as mean ± SD. NC: noncontaminated soil.

**Table 2:** Spearman correlation coefficient of environmental substrates on bacterial communities.

	Methylophilaceae*_Unclassified*	*Methylobacillus*	*Arthrobacter*	MB-A2-108*_Norank*	Vicinamibacterales*_Norank*	Sphingomonadaceae*_Uncultured*	Muribaculaceae*_Norank*
Ni	0.100	−0.219	−0.058	0.236	0.095	−0.290	−0.112
Cu	0.107	0.132	−0.266	−0.152	−0.157	0.250	0.363 *
Cr	−0.338	0.294	−0.285	−0.426 *	−0.377 *	0.406 *	0.402 *
Zn	−0.345	0.259	−0.246	−0.367 *	0.097	0.574 **	0.312
Cr (VI)	−0.444 *	0.487 **	−0.278	−0.500 **	−0.416 *	0.456 *	0.458 **
pH	0.558 **	−0.167	0.404 *	0.444 *	0.393 *	−0.154	−0.365 *

* *p* < 0.05, ** *p* < 0.01.

## Data Availability

The data presented in this study are available on request from the corresponding author.

## Supplementary Materials

The following are available online at https://www.mdpi.com/2076-2607/9/2/362/s1, Figure S1: The location of sampling sites; Figure S2: Rarefaction curves for the number of OTUs with more than 97% similarity threshold; Table S1: The soil PH and enzyme activities with different sampling sites; Table S2: The soil α-diversity with different sampling sites.
