# Supplementary material for: How the Soil Microbial Communities and Activities Respond to Long-Term Heavy Metal Contamination in Electroplating Contaminated Site
Source: Microorganisms. 2021 Feb 12;9(2):362. doi: 10.3390/microorganisms9020362 (PMC7918637; doi:10.3390/microorganisms9020362)
Supplement: Supplementary file 1 [file microorganisms-09-00362-s001.pdf]

Supporting Information for

How the soil microbial communities and activities response to long-term heavy metal contamination in electroplating contaminated site

Wen-Jing Gong[1], Zi-Fan Niu[1], He-Ping Zhao[1*]

1. MOE Key Lab of Environmental Remediation and Ecosystem Health, College of Environmental and Resource Sciences, Zhejiang University, Hangzhou, Zhejiang, China.

Table S1. The soil PH and enzyme activities with different sampling sites.

site	MBC (mg/kg)	UA (U/g)	PHA (U/g)	DHA (U/g)	pH
S0-1	69.928	0.2154	0.0612	0.0098	8.79
S0-2	115.963	0.4192	0.2453	0.0259	8.79
S0-3	99.676	0.4539	0.3617	0.0260	8.96
S0-4	94.602	0.3902	0.3311	0.0158	8.92
S0-5	26.762	0.3574	0.3188	0.0235	8.9
S1-1	74.987	0.0573	0.0503	0.0246	8.91
S1-2	120.397	0.0769	0.0719	0.0243	8.81
S1-3	54.859	0.0772	0.1042	0.0342	8.26
S1-4	105.014	0.0653	0.0395	0.0433	8.08
S1-5	29.126	0.1299	0.3832	0.0742	8.65
S2-1	23.939	0.0672	0.0179	0.0389	6.14
S2-2	22.920	0.0688	0.0069	0.0642	4.69
S2-3	30.834	0.0554	0.0178	0.1113	4.24
S2-4	88.080	0.0485	0.0180	0.2073	6.23
S2-5	31.854	0.0668	0.0182	0.0188	8.26
S3-1	86.901	0.0731	0.0500	0.0097	8.68
S3-2	12.124	0.0529	0.0394	0.0095	8.87
S3-3	61.353	0.0550	0.0287	0.0073	8.63
S3-4	55.432	0.2173	0.0830	0.0074	8.62
S3-5	77.284	0.4371	0.2232	0.0075	8.86
S4-1	58.798	0.1190	0.0506	0.0177	8.56
S4-2	89.693	0.0543	0.0180	0.0239	8.54
S4-3	83.892	0.0563	0.0289	0.0260	8.96
S4-4	59.096	0.0493	0.0071	0.0283	8.79
S4-5	132.535	0.0639	0.0398	0.0346	8.81
S5-1	139.263	0.3998	0.2764	0.0161	8.51
S5-2	91.854	0.1275	0.5341	0.0056	8.36
S5-3	87.887	0.2127	0.1263	0.0072	8.66
S5-4	78.068	0.0622	0.0505	0.0177	8.89
S5-5	38.924	0.0465	0.0288	0.0245	9.08
NC	187.922	0.2786	0.2656	0.0297	9.02

Note: NC: non-contaminated soil

Table S2. The soil α-diversity with different sampling sites.

site	chao	shannon	simpson
S0-1	1542	5.09	0.0294
S0-2	1361	3.01	0.1834
S0-3	1384	3.01	0.1973
S0-4	1142	5.33	0.0175
S0-5	1576	4.71	0.0588
S1-1	1008	2.89	0.3047
S1-2	725	2.49	0.2541
S1-3	542	2.31	0.2160
S1-4	227	2.59	0.2922
S1-5	453	1.09	0.6926
S2-1	122	3.28	0.0577
S2-2	889	4.29	0.0388
S2-3	63	2.92	0.0819
S2-4	601	4.67	0.0300
S2-5	622	3.75	0.1129
S3-1	1005	3.19	0.2724
S3-2	733	1.82	0.5308
S3-3	654	1.76	0.5275
S3-4	1243	5.14	0.0138
S3-5	1007	3.25	0.2178
S4-1	1000	4.37	0.0456
S4-2	705	3.20	0.1038
S4-3	928	3.88	0.0627
S4-4	1184	5.10	0.0168
S4-5	1138	4.30	0.0536
S5-1	1778	4.97	0.0633
S5-2	908	5.48	0.0080
S5-3	725	4.48	0.0476
S5-4	501	3.78	0.0945
S5-5	578	4.28	0.0509
NC	1467	5.87	0.0109

Note: NC: non-contaminated soil

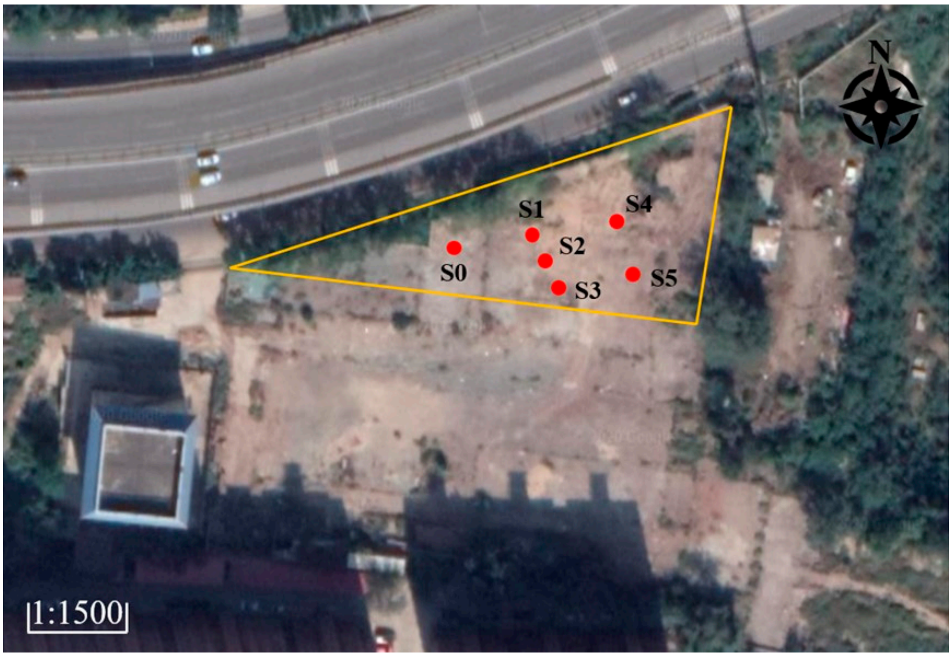

Figure S1. The location of sampling sites.

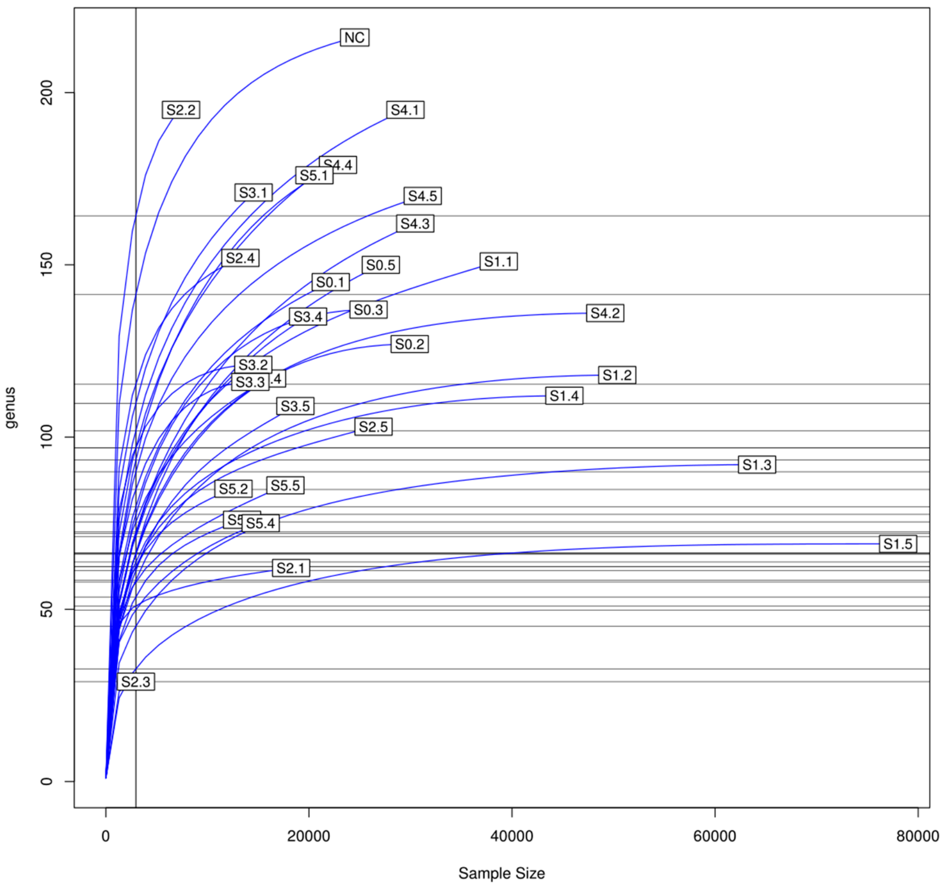

Figure S2. Rarefaction curves for the number of OTUs with more than 97% similarity threshold.

Abbreviated instructions

abbreviation	instruction
S0-1	electroplating laboratory 0.0-0.4m soil
S0-2	electroplating laboratory 0.4-0.8m soil
S0-3	electroplating laboratory 0.8-1.4m soil
S0-4	electroplating laboratory 1.4-2.2m soil
S0-5	electroplating laboratory 2.2-3.0m soil
S1-1	chrome bath 0.0-0.4m soil
S1-2	chrome bath 0.4-0.8m soil
S1-3	chrome bath 0.8-1.4m soil
S1-4	chrome bath 1.4-2.2m soil
S1-5	chrome bath 2.2-3.0m soil
S2-1	decorative chrome tank 0.0-0.4m soil
S2-2	decorative chrome tank 0.4-0.8m soil
S2-3	decorative chrome tank 0.8-1.4m soil
S2-4	decorative chrome tank 1.4-2.2m soil
S2-5	decorative chrome tank 2.2-3.0m soil
S3-1	post-plating treatment room 0.0-0.4m soil
S3-2	post-plating treatment room 0.4-0.8m soil
S3-3	post-plating treatment room 0.8-1.4m soil
S3-4	post-plating treatment room 1.4-2.2m soil
S3-5	post-plating treatment room 2.2-3.0m soil
S4-1	galvanizing bath 0.0-0.4m soil
S4-2	galvanizing bath 0.4-0.8m soil
S4-3	galvanizing bath 0.8-1.4m soil
S4-4	galvanizing bath 1.4-2.2m soil
S4-5	galvanizing bath 2.2-3.0m soil
S5-1	sewage treatment tank 0.0-0.4m soil
S5-2	sewage treatment tank 0.4-0.8m soil
S5-3	sewage treatment tank 0.8-1.4m soil
S5-4	sewage treatment tank 1.4-2.2m soil
S5-5	sewage treatment tank 2.2-3.0m soil
MBC	microbial biomass carbon
PHA	phosphatase
DHA	dehydrogenase
UA	urease
EAs	enzyme activities
HMs	heavy metals
NC	Non-contaminated soil
